# A Novel Educational Prescription Web-Based Application to Support Education for Caregivers of People Living With Dementia: Development and Usability Study With Clinicians

**DOI:** 10.2196/23904

**Published:** 2020-12-04

**Authors:** Anthony J Levinson, John Bousfield, William Douglas, Stephanie Ayers, Richard Sztramko

**Affiliations:** 1 Division of e-Learning Innovation McMaster University Hamilton, ON Canada; 2 McMaster University Hamilton, ON Canada; 3 GERAS Centre St. Peter's Hospital Hamilton, ON Canada

**Keywords:** dementia, caregiver, education prescription, online education, internet, eHealth, knowledge translation, implementation science, scale and spread

## Abstract

**Background:**

It is estimated that 564,000 Canadians are currently living with dementia and there are approximately 486,000 to 1.1 million informal family/friend caregivers. Family/friend caregivers often receive little to no education or training about dementia but are expected to provide ongoing support for a complex condition. Web-based family/friend caregiver interventions may be helpful, but little is known about how best to implement them.

**Objective:**

The objectives of this study were to 1) design and develop a novel education prescription application to help scale and spread web-based dementia education to family/friend caregivers, 2) conduct user testing, and 3) conduct a larger-scale field trial.

**Methods:**

A novel education prescription web-based application was designed and developed. Initial user testing used task completion and the “think aloud” technique with a small sample of representative clinicians who work with people living with dementia and family/friend caregivers. Following iterative incorporation of feedback, a larger field trial was conducted with a convenience sample of clinicians. Account invitations were sent to 55 clinicians and, following a 2-month trial period, surveys were administered to participants including the System Usability Scale and the Net Promoter Score.

**Results:**

During the initial user testing phase, participants (N=7) from representative disciplines easily completed associated tasks, and had very positive feedback with respect to the usability of the application. The System Usability Scale score during this phase was 91.4. Suggestions from feedback were incorporated into the application. During the larger field trial phase, participants (total N=55; activated account n=17; did not activate account n=38) were given access to the iGeriCare education prescription application. During this period, 2 participants created educational prescriptions; a total of 3 educational prescriptions were sent. Survey completers who did not activate their account (n=5) identified that their lack of use was due to time constraints, competing priorities, or forgetting to use the application. Survey completers who activated their account (n=5) identified their lower use was due to lack of time, lack of eligible family/friend caregivers during trial period, and competing priorities due to the COVID-19 pandemic. The System Usability Scale score during this phase was 78.75, and the Net Promoter Score was 50.

**Conclusions:**

Study findings indicate a generally positive response for the usability of a web-based application for clinicians to prescribe dementia education to family/friend caregivers. The dissonance between the promising data and widespread enthusiasm for the design and purpose of the education prescription application found in the initial user testing phase and subsequent lack of significant adoption in the field trial represents both an important lesson for other novel health technologies and a potential area for further investigation. Further research is required to better understand factors associated with implementation of this type of intervention and impact on dissemination of education to family/friend caregivers.

## Introduction

### Overview

Dementia is among the most prevalent long-term health conditions in Canada, with over 564,000 Canadians living with dementia and over 486,000 self-identified family/friend caregivers [1-4]. These numbers are predicted to increase substantially due to our aging population. In Canada, approximately 25,000 new cases of dementia are diagnosed each year; the number of people living with dementia is expected to increase by 66% to approximately 937,000 by 2031 [5].

Approximately 85% of people living with dementia rely on family/friend caregivers to provide support, despite the majority of these caregivers having no formal education or training related to the illness [6-8]. The Canadian National Dementia Strategy, Ontario Dementia Strategy, Health Quality Ontario Quality Standards for Dementia, and other clinical guidelines each highlight dementia caregiver education as an important component of quality care [3,4,9-12].

Web-based education for family/friend caregivers of people living with dementia has been shown to be effective for a number of caregiver outcomes. Several recent systematic reviews suggest web-based intervention programs have positive effects on self-efficacy, self-esteem, and strain of family/friend caregivers of adults living with a chronic condition [13-16]. There is also literature substantiating that web-based educational programs can benefit the mental health of family/friend caregivers for adults suffering from a chronic condition, particularly for the outcomes of caregiver depression, stress and distress, and anxiety [17,18]. These findings are particularly relevant for family/friend caregivers of people living with dementia, given the increased levels of distress and mental health conditions among family/friend caregivers [15,19]. Despite the evidence for the efficacy of web-based caregiver education, there are very few high-quality and freely-available programs available to family/friend caregivers of people living with dementia.

To respond to this unaddressed need, the award-winning iGeriCare online caregiver educational initiative was launched by a team of experts in online learning and dementia from McMaster University. iGeriCare contains multimedia lessons and resources, hosts live online events with content experts, and offers a series of microlearning emails to help educate family/friend caregivers of people living with dementia. iGeriCare targets caregivers through print-promotional materials (eg, clinic posters, print-based educational prescription pad, postcards), digital and social media marketing, and collaboration with community partners and intervention agents.

A qualitative study with dementia clinicians and other key stakeholders highlighted the utility of the educational prescription pad [20]. Participants reported that the educational prescription pad was a very efficient and effective way to direct family/friend caregivers to high-quality dementia education in the clinical setting [20]. When asked, participants agreed that an electronic version of the educational prescription concept would be potentially beneficial [20].

This work led to and informed the design and creation of a web-based educational prescription application. This application allows clinicians to electronically “prescribe” iGeriCare multimedia lessons to family/friend caregivers by sending an email to the caregiver with a link to a tailored curriculum of lessons.

### Objectives

In this paper, we describe 1) the design and development of the web-based education prescription application; 2) the initial user-testing phase, where we documented a small sample of clinicians’ initial impressions of the education prescription application; and 3) a field-trial phase, in order to better understand issues related to broader user acceptance and the feasibility of the education prescription application under real-world conditions.

## Methods

### Design and Development

The education prescription application is built using Laravel, an open source PHP framework that provides a solid foundation for web applications. The application is deployed on an Amazon Web Services EC2 instance and uses a MYSQL 8 database for data storage. This architecture allows for dynamic scaling and load balancing according to demand.

The development team, consisting of a project lead and a single full stack developer, met weekly to keep development focused and on track. A model-view-controller design pattern was employed to organize the application’s information structures. The front-end presentation is based on a clean Material Design approach in order to keep the user experience (ie, UI/UX) simple and intuitive.

Email notifications to family/friend caregivers are dispatched using a RESTful API from Mailgun, a third-party service that allows tracking of typical mail events such as opens, link clicks, and bounces. These email links are tokenized for additional tracking, and they direct the caregiver to prescribed content on the iGeriCare website.

Core refinements to functionality and the overall user experience evolved from discussions with other team members, informed by opinion and evidence from the initial user testing and qualitative interviews. Additional administrative and management features were added based on input from user testing and the field trial.

### Initial User Testing

A convenience sample of 7 representative health care providers who work with family/friend caregivers of people living with dementia were invited to provide feedback. This sample size has been shown to be relatively efficient and effective for usability testing [21]. The participants were recruited via email from healthcare providers who had previously voiced an interest in the education prescription application concept, while attempting to include some representation from diverse specialties and organizations (eg, family medicine, psychiatry, community advocacy organizations). User testing was carried out by a research assistant with extensive experience conducting participant interviews and a student from McMaster University’s Bachelor of Health Sciences program. The user testing took place in the participants’ workplace setting from November 5th, 2019 to January 14th, 2020. Participants accessed the education prescription application using their preferred personal devices, which in every case was either a desktop or laptop computer.

The usability testing protocol was derived from Krug’s usability testing methodology, namely the “think aloud” method [22]. This protocol was used with a focus on “task completion”.

Participants opened an invitation email to the education prescription application before being presented with a series of tasks to conduct within the application interface without outside assistance. Tasks included the following: open account invitation and activate, create profile, review dashboard, create educational prescription, review prescription metrics, and log out.

These tasks were chosen to reflect situations that users would encounter during normal use. After the task completion was complete, participants engaged in a brief semistructured interview and completed the 10-item System Usability Scale (see Multimedia Appendix 1). The System Usability Scale, an industry standard survey, is a brief but useful tool for obtaining reliable and valid results from usability tests with small sample sizes [23]. The feedback of participants from the “think aloud” method during task completion and subsequent semistructured interviews was recorded in writing and synthesized.

Feedback from the initial user testing was then incorporated into an updated beta version of the application, which was used for the field trial.

### Field Trial

Clinicians or organizations that had previously expressed interested in pilot testing the education prescription application or were recruited from a family medicine medical conference were invited via email to participate in the field trial. The goal was to achieve a minimum of at least 25 participants from a range of representative clinical disciplines/specialties and organizations working with family/friend caregivers of people living with dementia. In total, 55 participants were invited to participate. These participants had accounts created for them to use the fully functional beta version of the education prescription application. The field trial took place from February 4th, 2020 to June 1st, 2020.

The field trial was conducted with a mixed methods approach consisting of several distinct sources of data. The field trial period was defined as beginning immediately upon participant account creation and ending either after their 10th prescription or 2 months after account creation. Participants who were invited to the field trial and were inactive after 2 weeks were sent a reminder email to activate their account. Throughout the field trial, participants had the opportunity to provide written feedback through the education prescription application. At the end of the field trial, participants who activated their account were sent a “completer’s survey,” and those who did not activate their account were sent a “non-completer’s survey” using SurveyMonkey. Additional data was also collected at the end of the field trial, including the System Usability Scale ratings, Net Promoter Score, and utilization metrics recorded on the education prescription application website and through the application database. Net Promoter Score is a management tool that can be used to gauge customer satisfaction [24-26]. Using an 11-point scale, the Net Promoter Score asks respondents their “likelihood to recommend” a product or service based on their experience. The Net Promoter Score classifies respondents as either “detractors” (rated 0-6), “passives” (rated 7-8), or “promoters” (rated 9-10) and calculates the percentage of respondents in each group [26]. The percentage of detractors is then subtracted from the percentage of total promoters to give the final Net Promoter Score. Net Promoter Scores can range from -100 to +100.

The Hamilton Integrated Research Ethics Board reviewed the protocol and determined the study was quality improvement. The study was granted an exemption from full review.

## Results

### Education Prescription Application Description

**Figure 1:**
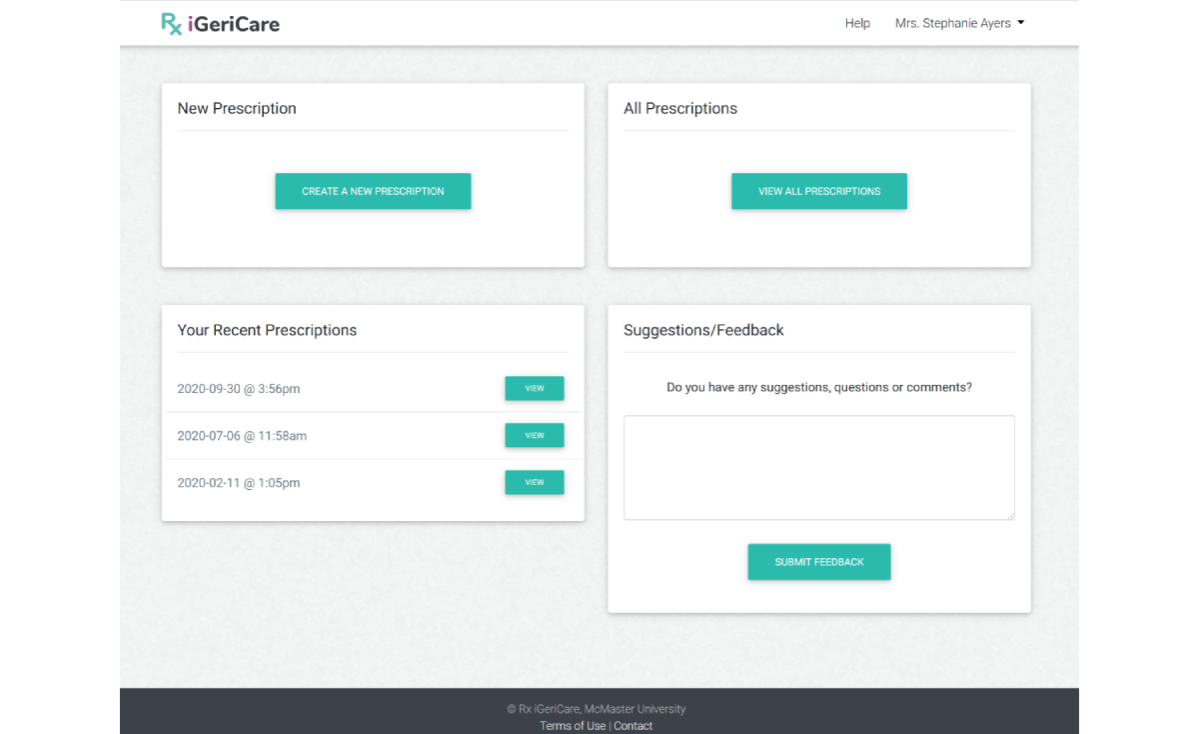
Education prescription application dashboard screen.

**Figure 2:**
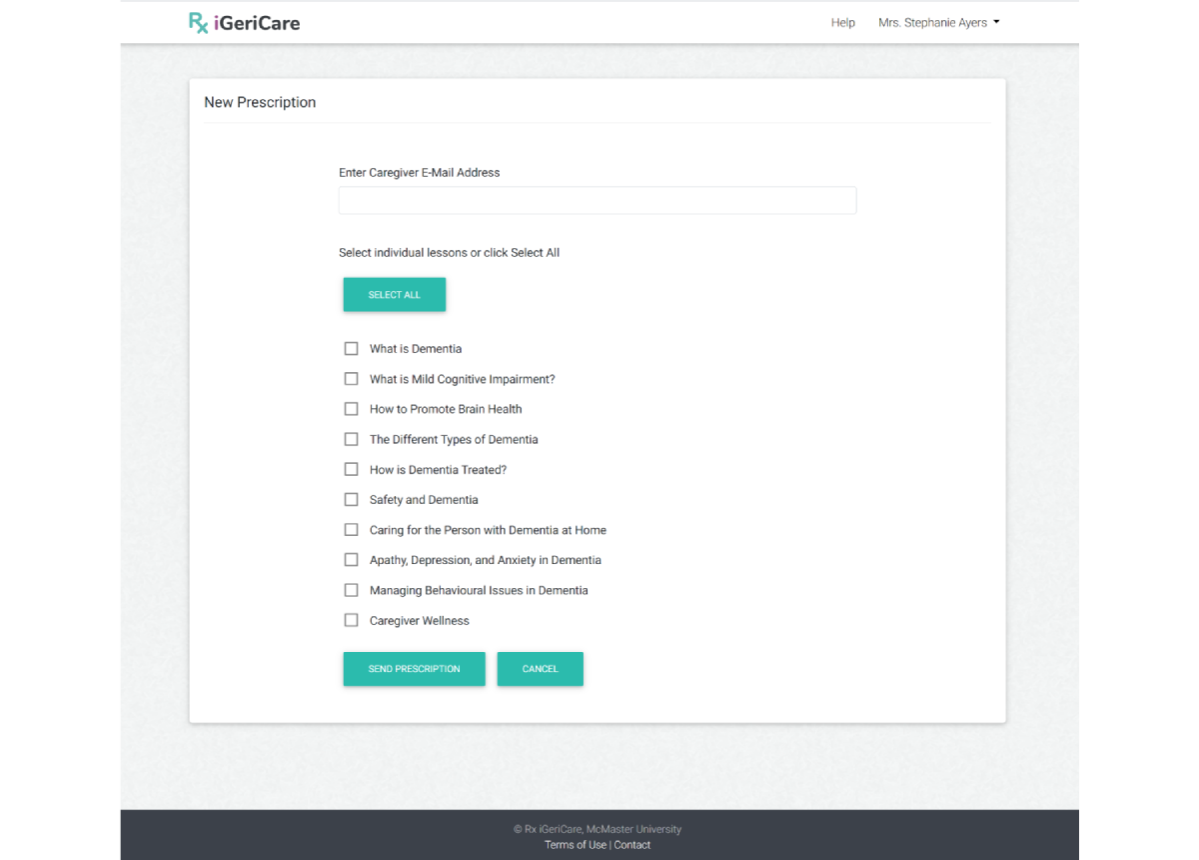
Education prescription application screen to create a new prescription.

Upon accessing the education prescription web application URL, the user is presented with a *login screen* which they can login to with the email and password that were used to create their account. Upon logging in, the user is directed to the *dashboard* (see Figure 1), which allows the user to navigate to and use the features of the application, such as *making a new prescription* (see Figure 2), *tracking their previous prescriptions*, or *giving feedback* on the application. These functions are all accessed by directing the user to a new page that allows them to complete the relevant task. Additionally, all pages after login have a banner at the top including the iGeriCare logo, which, if clicked, will bring the user back to the home page. All pages after login also have menu items to allow for the editing of account profile information and to request help/technical support.

### Initial User Testing

The initial user testing phase involved 7 user testers. Participants were located in Hamilton, Ontario and were from the following disciplines: family medicine (n=2), neurology (n=1), psychiatry (n=1), geriatrics (n=1), and community advocacy organization (n=2). The average System Usability Scale score for all initial user testers was 91.4 (N=7), with an average score among clinicians of 91.0 (n=5) and that of the community organization user testers being 92.5 (n=2). The System Usability Scale score of a typical application is 68, with a score of 80.3 representing the 90th percentile [23]. This places the education prescription application’s overall and subgroup scores within the 99th percentile of scores, which indicates a highly intuitive, well-designed, and functional application.

This data is supported by the feedback received in the semistructured interviews with initial user testers. The users overwhelmingly praised the logical design, ease-of-use, minimalist aesthetic, and utility of the education prescription application. All initial testers agreed that the term “educational prescription” was a descriptive and correct name for the application, and that it had the potential to give added legitimacy to the emails in the eyes of family/friend caregivers. Participants identified that they would like to know the password requirements at the account creation stage. Following the initial user testing phase, developers implemented this feedback and included password requirements on the account creation screen.

### Field Trial

Fifty-five participants were registered for the field trial phase, which included physicians and nonphysicians that respectively represented numerous specialties and roles, a mix of both urban and rural regions, as well as different Canadian provinces. Participants were recruited predominantly from a family medicine medical conference and from physicians who had been approached previously about the education prescription application. Due to the nature of our field trial recruitment strategy, we have limited information on the background of most of the participants. Participant demographics can be seen in Table 1.

Although 17 participants activated their accounts, only 2 participants created educational prescriptions. Fifty-three participants did not send any prescriptions during their field trial phase. A total of 3 educational prescriptions were sent; one participant sent 1 educational prescription, and another sent 2 educational prescriptions. No participants submitted written feedback through the education prescription application dashboard.

Of the 55 invited participants, 10 responses were received for the posttrial survey which included responses from 5 participants that activated their account and 5 nonactivators.

**Table 1:** Field trial participant demographics (N=55).

Description		Activators (n=17), n	Nonactivators (n=38), n
**Physician disciplines**			
	Geriatrician	2	—^a^
	Family medicine	7	—
**Other roles (exclusively long-term care staff)**			
	Manager	4	—
	Education	9	20
	Administration	1	—
	Human resources	1	—
	Unknown	—	18
**Province**			
	Ontario	12	20
	Alberta	3	—
	Manitoba	1	—
	Unknown	1	18
**Region characteristics**			
	Urban	15	20
	Rural	1	18
	Unknown	1	—

^a^Not applicable

#### Nonactivator Survey

There were 5 respondents for this survey; each respondent completed all questions. Participants were allowed to select multiple responses for each question. There were 3 responses that identified the primary reason for lack of use of the application was that they were “too busy.” Another 3 responses identified that they “forgot to use the app.” When asked if there were any changes that could be made to encourage the use of the application, 2 responses said “no,” 1 response said that they were “too busy for any change to have affected [their] action,” and 2 responses said that additional email reminders to activate their account may have been useful. One response said they “could not comment due to [their] lack of familiarity with the app.”

#### Activator Survey

There were 5 respondents for this survey; one of these respondents only completed the first question and did not go on to complete the remainder of the survey. Again, participants were allowed to select multiple responses for each question. When asked about reasons for low use of the application, the most popular responses were similar to those among nonactivators. Two responses identified that they were “too busy,” 2 responses identified “they forgot,” and an additional 2 responses said they “saw no dementia care partners during the field trial period.” It is interesting to note that one response specifically identified the COVID-19 pandemic as a reason for their low use of the education prescription application. Respondents did not identify anything that could be done differently with the education prescription application; one response highlighted that they were a “big fan of the tool,” and encouraged continued use of the application. Respondents all identified that they would like to use this application more frequently. The System Usability Scale score for the activator survey was 78.75, which falls within the “good” and “excellent” categories. The Net Promoter Score was 50, which is considered “excellent.”

## Discussion

### Principal Findings

In this study, we designed and developed a novel educational prescription web application for clinicians to efficiently prescribe iGeriCare multimedia lessons to family/friend caregivers of people living with dementia. Initial user testing validated the design and usability of the application, with very positive feedback on the application’s user-friendliness and functionality. The field trial was designed to look at real-world feasibility, usability, and function rather than broader implementation issues or scale and spread. Despite initial user testing validation, during the field trial most participants did not use the application at all, and those who used it wrote very few prescriptions. Feedback from participants suggests that this was due to generally being too busy and not a function of the application itself. Timing of the field trial overlapped with the COVID-19 pandemic, which also had a substantial impact on use during the field trial. Nonetheless, participants voiced positive comments and enthusiasm for the application. To our knowledge, this is the first study describing the design, development, and usability testing of this type of novel application for educational prescriptions.

### Nonpharmacological Prescriptions and Information Provision

There is very little evidence surrounding the success or value of nonpharmacological prescriptions, such as the “social prescribing model” [27]. Research on “information provision” more generally also suffers from a lack of high quality research that could inform the implementation of the education prescription application; however, there are some systematic reviews that indicate some general trends, such as the positive psychological effects of disease knowledge on people living with dementia and family/friend caregivers, and practical skills-based education’s stronger association with positive health outcomes [28,29]. Additionally, literature exploring the efficacy of multimedia information provision compared to print information and verbal education by healthcare professionals tends to indicate it is as, or more, effective than these methods, justifying the chosen medium for iGeriCare’s online learning [28].

### Diffusion of Innovation

Another theoretical framework relevant to the field of healthcare technology implementation is the “diffusion of innovation” theory, which describes “how, why, and at what rate new ideas and technology spread” [30,31]. This theory pioneered the concept of early adopters being the critical force in driving widespread acceptance of novel technologies, policies, and ideas, and has been successfully applied and adapted to health and information technologies by major institutions such as the National Health Service [32,33]. Wainwright and Waring note that professionally dominated organizational cultures tend to rely strongly on authority adoption decisions for effective uptake of novel information technology [33]. This effect was found to scale with the size of the organization adopting a technology, with larger organizations such as healthcare systems and hospital networks having more difficulty adopting technology without official sanction. The limited uptake by independent physicians observed in the field trial despite positive user-testing feedback on the application itself may be explained by this mechanism, given the novel and voluntary nature of education prescription application use.

### Barriers to eHealth Technology Adoption

The findings of this study are also relevant to the field of implementation science, especially relating to healthcare technology. The NASSS framework (designed to evaluate nonadoption, abandonment, and challenges to the scale-up, spread, and sustainability of health and care technologies) is an evidence-based, theory-informed, and pragmatic framework describing seven domains of healthcare technology, and how simplicity or complexity in these domains effects the likelihood of successful implementation and proliferation [34]. The NASSS framework consists of a series of questions in 7 domains: (1) the condition (or illness), (2) the technology, (3) the value proposition, (4) the adopter system (staff, patient, and lay caregivers), (5) the organization, (6) the wider context (institutional and societal), and (7) the interaction and mutual adaptation between all domains over time [34]. Investigating the 7 domains in reference to the education prescription application further substantiates that the *technology*, *value*, *organizations*, *wider system*, and *adaptability* of the education prescription application avoid complexity and facilitate implementation [35].

Some potential complexity arises in the “condition” domain, as “dementia” is an umbrella term for a syndrome caused by several different disorders, with highly variable symptom severity and rates of progression [36]. Moreover, as a condition that typically affects older adults, many of the application’s eventual prescription recipients (eg, the family/friend caregivers) may also be older adult spousal caregivers. Based on our earlier qualitative research, some clinicians still view older adults as reluctant to use email- or web-based technologies, despite evidence to the contrary [20]. Those clinician attitudes may have dissuaded them from using the education prescription application with older family/friend caregivers. In the “adopter system” domain, our earlier qualitative research had also identified physician reluctance to use any additional electronic prescribing tool that was not integrated directly with their electronic medical records and clinical workflows [20].

A recent scoping review on the adoption of eHealth technology by physicians identified several barriers to the adoption and implementation of eHealth technologies that may be relevant to understanding low usage of the education prescription application during the field trial. Studies have identified the lack of harmonization of eHealth systems as a notable barrier, consistent with our qualitative research with respect to physicians wanting the application integrated with their electronic medical records systems [20,37]. While not voiced by participants in our field trial specifically, privacy and security concerns may have played a role with respect to low usage, as physicians are unaccustomed to using email to send messages to people living with dementia or their families. Lack of time and workload were also identified in this scoping review, which would be consistent with participant feedback during our qualitative interviews.

### Conclusions

This study highlights an interesting tension or gap between positive usability feedback and actual use of novel information technologies in a healthcare setting. In particular, the dissonance between the promising data and widespread enthusiasm about the design and purpose of the education prescription application found in the initial user testing phase and subsequent lack of significant adoption in the field trial represents both an important lesson for other novel health technologies and a potential area for further investigation. The timing of the onset of the COVID-19 pandemic was likely an important factor for the low adoption by participants. Had our trial began a few months *after* the onset of the pandemic, it is possible that we might have had greater uptake from individual participants as well as organizations. In the first month of the pandemic, there was initially a lot less clinical activity due to competing priorities stemming from the pandemic; however, after the first month or two, there was increasing and widespread interest in virtual tools and virtual education. An additional impact caused by the COVID-19 pandemic was the fact that some clinicians saw no family/friend caregivers of people living with dementia during the trial period.

Future field trials for the iGeriCare education prescription application will focus on implementation settings with high volumes of family/friend caregivers of people living with dementia such as dementia clinics, memory clinics, long-term care home organizations, and community dementia advocacy organizations, which will allow us to further our understanding of the most probable implementation settings.

## Appendix

Multimedia Appendix 1 System usability scale (SUS).

## Abbreviations

NASSS: non-adoption, abandonment, scale-up, spread, sustainability
