# Supplementary material for: A Novel Educational Prescription Web-Based Application to Support Education for Caregivers of People Living With Dementia: Development and Usability Study With Clinicians
Source: JMIR Hum Factors. 2020 Dec 4;7(4):e23904. doi: 10.2196/23904 (PMC7748956; doi:10.2196/23904)
Supplement: Multimedia Appendix 1 [file humanfactors_v7i4e23904_app1.pdf]

Participant ID: _____ Site: __________________________ Date: ___/___/___

System Usability Scale

Instructions: For each of the following statements, mark <u>one</u> box that best describes your reactions to the website *today*.

		Strongly Disagree				Strongly Agree
1.	I think that I would like to use this website frequently.	☐	☐	☐	☐	☐
2.	I found this website unnecessarily complex.	☐	☐	☐	☐	☐
3.	I thought this website was easy to use.	☐	☐	☐	☐	☐
4.	I think that I would need assistance to be able to use this website.	☐	☐	☐	☐	☐
5.	I found the various functions in this website were well integrated.	☐	☐	☐	☐	☐
6.	I thought there was too much inconsistency in this website.	☐	☐	☐	☐	☐
7.	I would imagine that most people would learn to use this website very quickly.	☐	☐	☐	☐	☐
8.	I found this website very cumbersome/awkward to use.	☐	☐	☐	☐	☐
9.	I felt very confident using this website.	☐	☐	☐	☐	☐
10.	I needed to learn a lot of things before I could get going with this website.	☐	☐	☐	☐	☐

Please provide any comments about this website:

This questionnaire is based on the System Usability Scale (SUS), which was developed by John Brooke while working at Digital Equipment Corporation. © Digital Equipment Corporation, 1986.
